# CAAC Boranes. Synthesis and characterization of cyclic (alkyl) (amino) carbene borane complexes from BF_3_ and BH_3_

**DOI:** 10.3762/bjoc.6.82

**Published:** 2010-08-02

**Authors:** Julien Monot, Louis Fensterbank, Max Malacria, Emmanuel Lacôte, Steven J Geib, Dennis P Curran

**Affiliations:** 1 UPMC Univ. Paris 06, Institut parisien de chimie moléculaire (UMR CNRS 7201), C 229, 4 place Jussieu, 75005 Paris, France; 2 Department of Chemistry, University of Pittsburgh, Pittsburgh, PA 15260, USA

**Keywords:** borane Lewis acid Lewis base complexes, carbene–borane complexes, cyclic (alkyl) (amino) carbenes, *N*-heterocyclic carbenes, stable carbenes

## Abstract

In situ formation of two cyclic (alkyl) (amino) carbenes (CAACs) followed by addition of BF_3_•Et_2_O provided the first two examples of CAAC–BF_3_ complexes: 1-(2,6-diisopropylphenyl)-3,5,5-trimethyl-3-phenylpyrrolidin-2-ylidene trifluoroborane, and 2-(2,6-diisopropylphenyl)-3,3-dimethyl-2-azaspiro[4.5]decan-1-ylidene trifluoroborane. These CAAC–BF_3_ complexes are robust compounds that are stable to ambient laboratory conditions and silica gel chromatography. They were characterized by spectroscopy and X-ray crystallography. In contrast, a CAAC complex with borane (BH_3_) was readily formed in situ according to ^1^H and ^11^B NMR analysis, but did not survive the workup conditions. These results set the stage for further studies of the chemistry of CAAC boranes.

## Introduction

**Figure 1:**
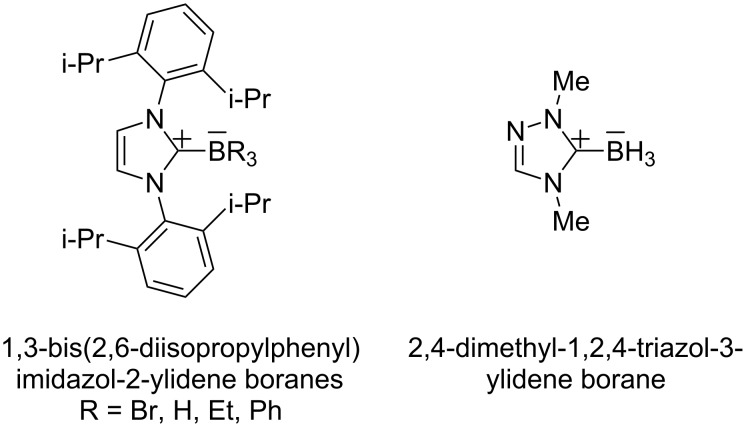
Representative complexes of *N*-heterocyclic carbenes and boranes (NHC–boranes).

Lewis acid/Lewis base complexes of *N*-heterocyclic carbenes and boranes (NHC–boranes) are readily prepared from NHC’s and boranes by direct complexation [1–4]. Unlike many other classes of Lewis base complexes of boranes with neutral molecules (ethers, sulfides, etc.), NHC–borane complexes are highly stable in diverse environments. Complexes such as those shown in Figure 1 are white solids that can often be chromatographed if desired. Many such complexes resist decomplexation, oxidation, and both acidic and basic hydrolysis. Such NHC–boranes are beginning to be used as synthetic reagents, with applications in radical [5–9], ionic [10–11] and organometallic [12] reactions. They are also precursors for making higher complexes with unusual bonding patterns such as boron–boron double bonds [3], or for making unusual reactive intermediates on boron [6–7,13].

**Figure 2:**
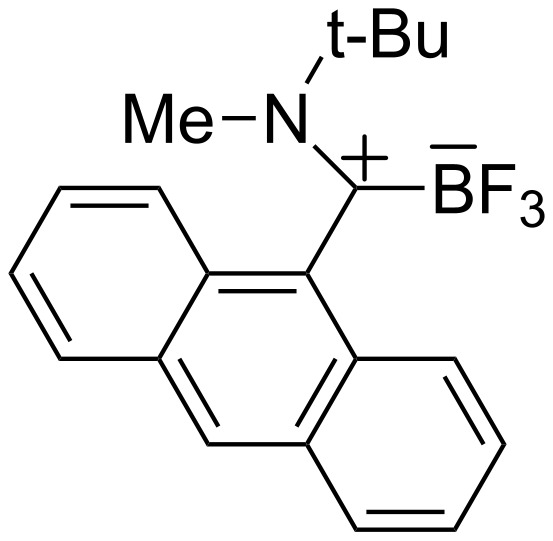
Bertrand’s amino anthracenyl carbene trifluoroborane complex.

Most of the first generation carbene-boranes have been made from *N*-heterocyclic carbenes in which the carbene carbon is stabilized by two donating nitrogen atoms (imidazolylidene, triazolylidene, etc) or other heteroatoms (phosphorous, silicon, oxygen) [14–18]. An exception is Bertrand’s BF_3_ complex of an acyclic (amino) (aryl) carbene in which the aryl group is 9-anthracenyl [19]. This complex, whose structure is shown in Figure 2, has also two cation stabilizing groups on the carbene carbon.

Accordingly, it is interesting to study borane complexes of other types of carbenes. Recently, Bertrand introduced, cyclic (alkyl) (amino) carbenes, or CAACs, as a new class of carbene complex in which the central carbene carbon is stabilized by only one nitrogen atom [20–23]. Herein we report the synthesis and characterization (including X-ray structures) of two stable CAAC–BF_3_ complexes. We also show through spectroscopic studies that a representative CAAC–BH_3_ complex can be generated in solution.

## Results and Discussion

**Scheme 1:**
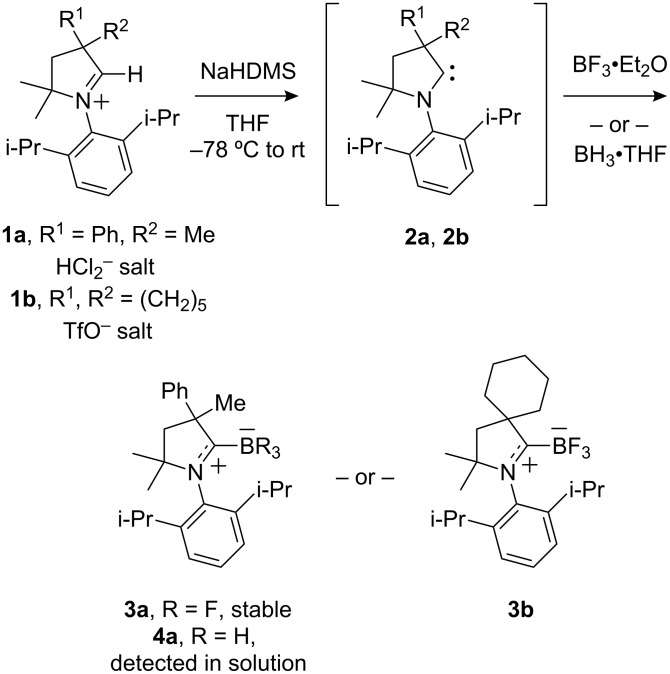
Synthesis of stable CAAC–BF_3_ complexes **3a** and **3b** and in situ generation of CAAC–BH_3_ complex **4a**.

CAAC trifluoroborane complexes **3a** and **3b** were readily prepared as summarized in Scheme 1. Deprotonation of the 3,4-dihydro-*2H*-pyrrolium hydrogen dichloride salt **1a** with NaHMDS in THF at −78 °C, followed by warming to room temperature gave a pale yellow solution of the free carbene **2a** [20]. A broad resonance at −312 ppm in the ^13^C NMR spectrum of this solution indicated that **2a** had formed. Boron trifluoride etherate (BF_3_•Et_2_O) was then added at −78 °C and the resulting solution was stirred overnight. Rapid filtration through silica gel afforded the pure complex **3a** (1-(2,6-diisopropylphenyl)-3,5,5-trimethyl-3-phenylpyrrolidin-2-ylidene trifluoroborane) as white crystals in 61% yield. CAAC borane **3b** (2-(2,6-diisopropylphenyl)-3,3-dimethyl-2-azaspiro[4.5]decan-1-ylidene trifluoroborane) was made by a similar procedure starting from triflate salt **1b** and was isolated in 64% yield.

**Figure 3:**
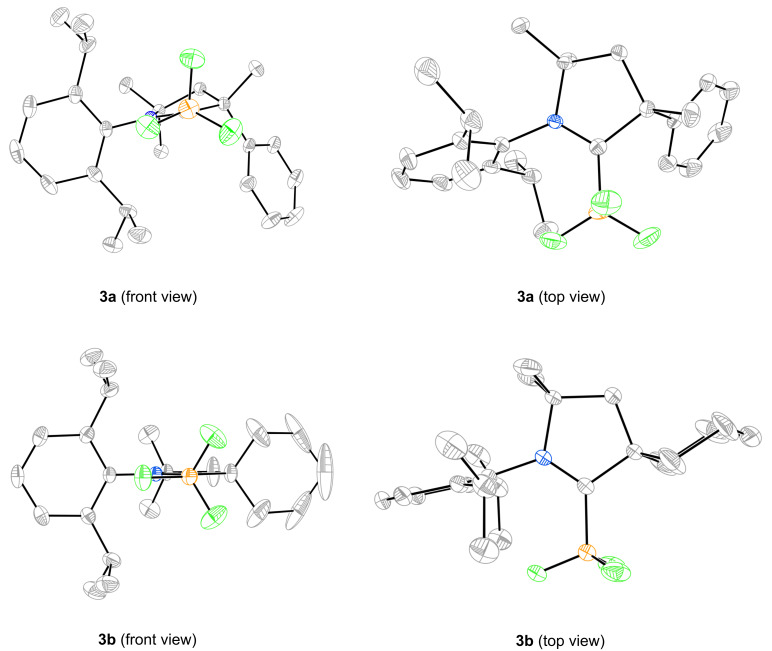
X-Ray crystal structures of CAAC–BF_3_ complexes **3a** (top) and **3b** (bottom).

CAAC boranes **3a** and **3b** were obtained as white crystals, and the structure of a single crystal of each was solved by X-ray diffraction. Two views of each structure are shown in Figure 3. Of special interest are the lengths of the C_carbene_–N bonds. At 1.294(3) Å for **3a** and 1.293(3) Å for **3b**, these bonds are significantly shorter than analogous bonds on other NHC–BF_3_ complexes where the carbene carbon has two nitrogen substituents (1.338(5)–1.355(5) Å) [24–25]. Instead, these bond lengths are closer to that of the BF_3_ complex of the amino-anthryl-carbene (1.301(2) Å) shown in Figure 2 [19]. Presumably, this is because there is more demand on the lone nitrogen of CAAC complex to stabilize the positive charge than when two nitrogen atoms are present. In contrast, the C_carbene_–B bond lengths of **3a** (1.674(3) Å) and **3b** (1.681(3) Å) are somewhat longer than those in other NHC–BF_3_ complexes (1.635(5)–1.668(6) Å) [24–25].

To conclude this preliminary study, we briefly attempted formation of the borane complex **4a** by generation of carbene **2a** as above, followed by the addition of BH_3_•THF. Indeed, complex **4a** was directly formed in situ as evidenced by ^11^B NMR spectroscopy. The resonance for BH_3_•THF was absent and in its place was a new quartet at −30.0 ppm. Both the chemical shift and multiplicity of this resonance are consistent with the CAAC–borane structure **4a**. The ^1^H NMR spectrum of the reaction mixture also showed the formation of **4a** (see Supporting Information File 1).

The reaction mixture was allowed to stand for 15 h, during which time the ^11^B NMR spectrum was unchanged. This suggests that complex **4a** is thermally stable. However, standard workup and evaporation as used for NHC–boranes and CAAC–BF_3_ complexes **3a** and **3b** did not provide **4a**. Evidently, **4a** is not as stable towards isolation as the BH_3_ complexes shown in Figure 1. Nonetheless, it should still be possible to generate this complex in situ and use it directly for onward reactions.

## Conclusion

In summary, we have synthesized the first CAAC borane complexes. The complexes of trifluoroborane (BF_3_) are stable and were isolated as pure solids and fully characterized by spectroscopic analysis and X-ray crystallography. The borane (BH_3_) complex was characterized in situ by NMR spectroscopy, but did not survive workup and isolation. These results set the stage for further studies of the chemistry of CAAC boranes.

## Supporting Information

File 1 Procedures and characterization of the new complexes.

File 2 Cif file of crystal structure of compound **3a**.

File 3 Cif file of crystal structure of compound **3b**.

## References

[1] Kuhn N, Henkel G, Kratz T, Kreutzberg J, Boese R, Maulitz A H (1993). Chem Ber.

[2] Ramnial T, Jong H, McKenzie I D, Jennings M, Clyburne J A C (2003). Chem Commun.

[3] Wang Y, Quillian B, Wei P, Wannere C S, Xie Y, King R B, Schaefer H F, v. R. Schleyer P, Robinson G H (2007). J Am Chem Soc.

[4] Yamaguchi Y, Kashiwabara T, Ogata K, Miura Y, Nakamura Y, Kobayashi K, Ito T (2004). Chem Commun.

[5] Ueng S-H, Makhlouf Brahmi M, Derat É, Fensterbank L, Lacôte E, Malacria M, Curran D P (2008). J Am Chem Soc.

[6] Matsumoto T, Gabbaï F P (2009). Organometallics.

[7] Ueng S-H, Solovyev A, Yuan X, Geib S J, Fensterbank L, Lacôte E, Malacria M, Newcomb M, Walton J C, Curran D P (2009). J Am Chem Soc.

[8] Walton J C, Makhlouf Brahmi M, Fensterbank L, Lacôte E, Malacria M, Chu Q, Ueng S-H, Solovyev A, Curran D P (2010). J Am Chem Soc.

[9] Tehfe M-A, Makhlouf Brahmi M, Fouassier J-P, Curran D P, Malacria M, Fensterbank L, Lacôte E, Lalevée J (2010). Macromolecules.

[10] Chu Q, Makhlouf Brahmi M, Solovyev A, Ueng S-H, Curran D P, Malacria M, Fensterbank L, Lacôte E (2009). Chem–Eur J.

[11] Lindsay D M, McArthur D (2010). Chem Commun.

[12] Monot J, Makhlouf Brahmi M, Ueng S-H, Robert C, Desage-El Murr M, Curran D P, Malacria M, Fensterbank L, Lacôte E (2009). Org Lett.

[13] Braunschweig H, Chiu C-W, Radacki K, Kupfer T (2010). Angew Chem, Int Ed.

[14] Alcaraz G, Reed R, Baceiredo A, Bertrand G (1993). Chem Commun.

[15] Lambert C, Lopez-Solera I, Raithby P R (1996). Organometallics.

[16] Tamm M, Lügger T, Hahn F E (1996). Organometallics.

[17] Merceron N, Miqueu K, Baceiredo A, Bertrand G (2002). J Am Chem Soc.

[18] Snead D R, Ghiviriga I, Abboud K A, Hong S (2009). Org Lett.

[19] Cattoën X, Gornitzka H, Bourissou D, Bertrand G (2004). J Am Chem Soc.

[20] Lavallo V, Canac Y, Präsang C, Donnadieu B, Bertrand G (2005). Angew Chem, Int Ed.

[21] Lavallo V, Canac Y, DeHope A, Donnadieu B, Bertrand G (2005). Angew Chem, Int Ed.

[22] Jazzar R, Dewhurst R D, Bourg J-B, Donnadieu B, Canac Y, Bertrand G (2007). Angew Chem, Int Ed.

[23] Zeng X, Frey G D, Kinjo R, Donnadieu B, Bertrand G (2009). J Am Chem Soc.

[24] Arduengo A J, Davidson F, Krafczyk R, Marshall W J, Schmutzler R (2000). Monatsh Chem.

[25] Nielsen D J, Cavell K J, Skelton B W, White A H (2003). Inorg Chim Acta.

