# Supplementary material for: CAAC Boranes. Synthesis and characterization of cyclic (alkyl) (amino) carbene borane complexes from BF3 and BH3
Source: Beilstein J Org Chem. 2010 Aug 2;6:709–12. doi: 10.3762/bjoc.6.82 (PMC2956467; doi:10.3762/bjoc.6.82)
Supplement: File 1 — Procedures and characterization of the new complexes. [file Beilstein_J_Org_Chem-06-709-s001.pdf]

Supporting Information

for

CAAC Boranes. Synthesis and characterization of cyclic (alkyl) (amino) carbene borane complexes from BF_3 and BH_3

Julien Monot[1,2], Louis Fensterbank*[1], Max Malacria*[1], Emmanuel Lacôte*[1], Steven J. Geib[2] and Dennis P. Curran*[2]

[1]UPMC Univ. Paris 06, Institut parisien de chimie moléculaire (UMR CNRS 7201), C 229, 4 place Jussieu, 75005 Paris, France and

[2]Department of Chemistry, University of Pittsburgh, Pittsburgh, PA 15260 USA

Email: Julien Monot - jam287@pitt.edu; Louis Fensterbank* - louis.fensterbank@upmc.fr; Max Malacria* - max.malacria@upmc.fr; Emmanuel Lacôte* - emmanuel.lacote@upmc.fr; Steven J. Geib - geib@pitt.edu; Dennis Curran* - curran@pitt.edu

*Corresponding author

Table of Contents

General remarks S2
Experimental Procedures S2
NMR experiment: in situ generation of the CAAC–BH_3 **4a** S4

General Remarks. Chemicals and solvents were purchased from commercial suppliers and used as received. Reactions were carried out under an argon atmosphere, with magnetic stirring and redistilled solvents when necessary. Thin layer chromatography (TLC) was performed on Merck 60 F254 silica gel, Merck Geduran SI 60 A silica gel (35−70 mm) was used for column chromatography. Melting points were taken with a SMP3 Stuart Scientific melting point apparatus or with a Mel-Temp II apparatus and are uncorrected. IR spectra were recorded with a Bruker Tensor 27 ATR diamant PIKE spectrometer or a Nicolet Avatar 360 FT-IR spectrometer as thin films (CH_2Cl_2) on NaCl plates. ^{1}H, ^{19}F, ^{13}C and ^{11}B NMR spectra were recorded with Bruker Avance 400 spectrometer fitted with a BBFO probe ^{19}F/^{13}C/^{11}B/^{1}H including z gradient. Chemical shifts are given in ppm. Unless otherwise noted, the NMR spectra were recorded in $CDCl_3$. Chloroform (δ = 7.26 ppm) was used as internal standard in ^{1}H NMR spectra, whereas $CDCl_3$ (δ = 77.2 ppm) was used as internal standard in ^{13}C NMR spectra. ^{11}B chemical shifts are relative to Et_2O•BF_3. ^{19}F chemical shifts are relative to $CFCl_3$. Coupling constants (*J*) are given in Hertz (Hz). Mutiplicities are denoted by m (multiplet), s (singlet), d (doublet), t (triplet), q (quartet), sept. (septet). 1-(2,6-Diisopropylphenyl)-2,2,4-trimethyl-1,4-diphenyl-3,4-dihydro-*2H*-pyrrolium hydrogen dichloride **1a** was kindly donated by Rhodia. 3,3-Dimethyl-2-(2,6-diisopropylphenyl)-2)azoniaspiro[4.5]dec-1-ene triflate **1b** was synthesized according the procedure described in the literature [1].

Experimental Procedures:

1-(2,6-Diisopropylphenyl)-3,5,5-trimethyl-3-phenylpyrrolidin-2-ylidene trifluoroborane 3a:

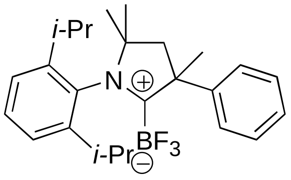

A solution of NaHMDS (1 M in THF, 2.2 equiv, 2.6 mL, 2.6 mmol) was added to a solution of iminium salt **1a** (0.5 g, 1.19 mmol) in THF (6 mL) at –78 °C. The suspension was warmed to room temperature and stirred for 1 h. Then, a solution of BF_3•Et_2O in diethyl ether (46% in Et_2O, 1.1 equiv, 0.45 mL, 1.3 mmol) was added at –78 °C. The reaction mixture was stirred overnight

and slowly warmed to room temperature. After evaporation of the solvent, the residue was purified by flash chromatography (hexane/AcOEt : 90/10) to afford a white solid (252 mg, 61%). mp 205-207 °C; IR (neat): ν = 3074, 2989, 2968, 2933, 2870, 1590, 1579, 1494, 1468, 1445, 1393, 1386, 1379, 1373, 1365, 1326, 1131, 1090, 1081, 1031, 943, 919, 814, 769 cm^{-1}; ^{1}H NMR (300 MHz, $CDCl_3$): δ 7.5-7.2 (m, 8H), 2.86 (sept., *J* = 6.6 Hz, 1H), 2.76-2.69 (m, 2H), 2.4 (d, *J* = 13.2 Hz, 1H), 2.02 (s, 3H), 1.40-1.26 (m, 15H), 1.19 (s, 3H); ^{13}C NMR (100 MHz, $CDCl_3$): δ 145.3, 144.9, 144.1, 132.3, 129.8, 128.4, 127.1, 126.6, 125.1, 124.7, 81.3, 59.3, 53.4, 29.4, 29.3, 28.6, 27.8, 26.6, 25.9, 25.8, 25.3, 24.1, 23.6; ^{11}B NMR (BF_3•Et_2O, 96.3 MHz, $CDCl_3$): δ -0.68 (q, $J_{B\text{-}F}$ = 38.1 Hz); ^{19}F NMR (376 MHz, $CDCl_3$) δ –137.2; HRMS calcd. For $C_{25}H_{33}{}^{11}BNF_3Na$: 438.2556, found 438.2566.

2-(2,6-Diisopropylphenyl)-3,3-dimethyl-2-azaspiro[4.5]decan-1-ylidene trifluoroborane 3b:

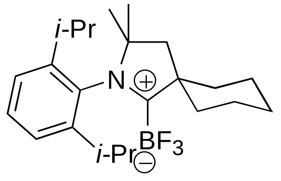

A solution of NaHMDS (1 M in THF, 2.2 equiv, 2.6 mL, 2.6 mmol) was added to a solution of iminium salt **1b** (0.5 g, 1.05 mmol) in THF (6 mL) at –78 °C. The suspension was warmed to room temperature and stirred for 1 h. Then, a solution of BF_3•Et_2O in diethyl ether (46% in Et_2O, 1.1 equiv, 0.3 mL, 1.16 mmol) was added at –78 °C. The reaction mixture was stirred overnight and slowly warmed to room temperature. After evaporation of the solvent, the residue was purified by flash chromatography (hexane/AcOEt : 90/10) to afford a white solid (265 mg, 64 %). mp 250 °C (decomp.); IR (neat): ν = 2973, 2930, 2350, 1578, 1471, 1446, 1389, 1369, 1331, 1317, 1081, 1060, 990, 971, 929, 814 cm^{-1}; ^{1}H NMR (400 MHz, $CDCl_3$): δ 7.41 (t, *J* = 8.1 Hz, 1H), 7.26 (d, *J* = 7.5 Hz, 2H), 2.66 (sept., *J* = 6.6 Hz, 2H), 2.6-2.4 (m, 2H), 2.21 (s, 2H), 1.86-1.7 (m, 3H), 1.66-1.61 (m, 2H), 1.5-1.4 (m, 3H), 1.38 (s, 6H), 1.31 (d, *J* = 6.6 Hz, 6H), 1.23 (d, *J* = 6.6 Hz, 6H); ^{13}C NMR (100 MHz, $CDCl_3$): δ 144.4, 132.1, 129.6, 124.5, 80.6, 57.7, 45.1, 34.0, 29.3, 29.2, 25.5, 24.8, 23.5, 21.9; ^{11}B NMR (BF_3•Et_2O, 96.3 MHz, $CDCl_3$): δ -0.66 (q, $J_{B\text{-}F}$ = 39.5 Hz);^{19}F NMR (376 MHz, $CDCl_3$) δ –138.0; HRMS calcd. For $C_{23}H_{35}{}^{11}BNF_3Na$: 416.2712, found 416.2698.

NMR experiment: in situ generation of the CAAC-BH_3 4a:

Before the experiment, a ^{1}H NMR spectrum of **1a** in THF-d_8 was recorded as a standard. The progress of the reactions was followed by changes in the chemical shifts of the CH_2 (grey spots) and the CH (pink spots) resonances (See Scheme S1 and Figure S2 spectrum **A**). Then, a solution of NaHMDS (1 M in THF, 2.2 equiv, 2.6 mL, 2.6 mmol) was added to the solution of iminium salt **1a** (0.5 g, 1.19 mmol) in THF-d_8 (6 mL) at –78 °C. The suspension was warmed to room temperature and stirred (See Scheme S2). After 1 h, a ^{1}H NMR spectrum was recorded and the new resonances of the CH_2 (green spots) and the CH (blue spots) for **B** were observed (See Figure S2 spectrum **B**). Then, a solution of BH_3•THF (1 M in THF, 1.1 equiv, 1.3 mL, 1.3 mmol) was added at –78 °C. The reaction mixture was slowly warmed to room temperature and stirred for 2.5 h. At this point, ^{1}H and ^{11}B NMR spectra were recorded. The ^{11}B NMR chemical shift and multiplicity, a quartet at –30 ppm (See Figure S3), confirmed the *in situ* generation of the CAAC-BH_3 complex **C**, as did the new resonances in ^{1}H NMR for the CH_2 (yellow spots) and the CH (red spots). The ^{11}B NMR spectrum was unchanged after 15 h (See Figure S4), suggesting that the complex is reasonably thermally stable. However, these resonances disappeared from the sample after evaporative workup.

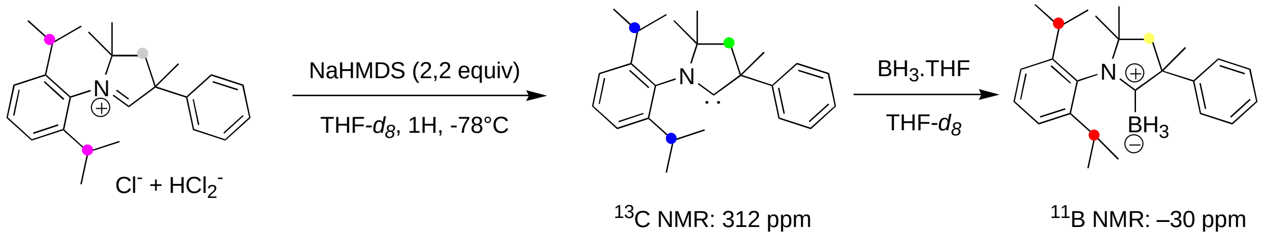

1a **2a** **4a**

Scheme S1: NMR experiment: in situ generation of the CAAC-BH_3

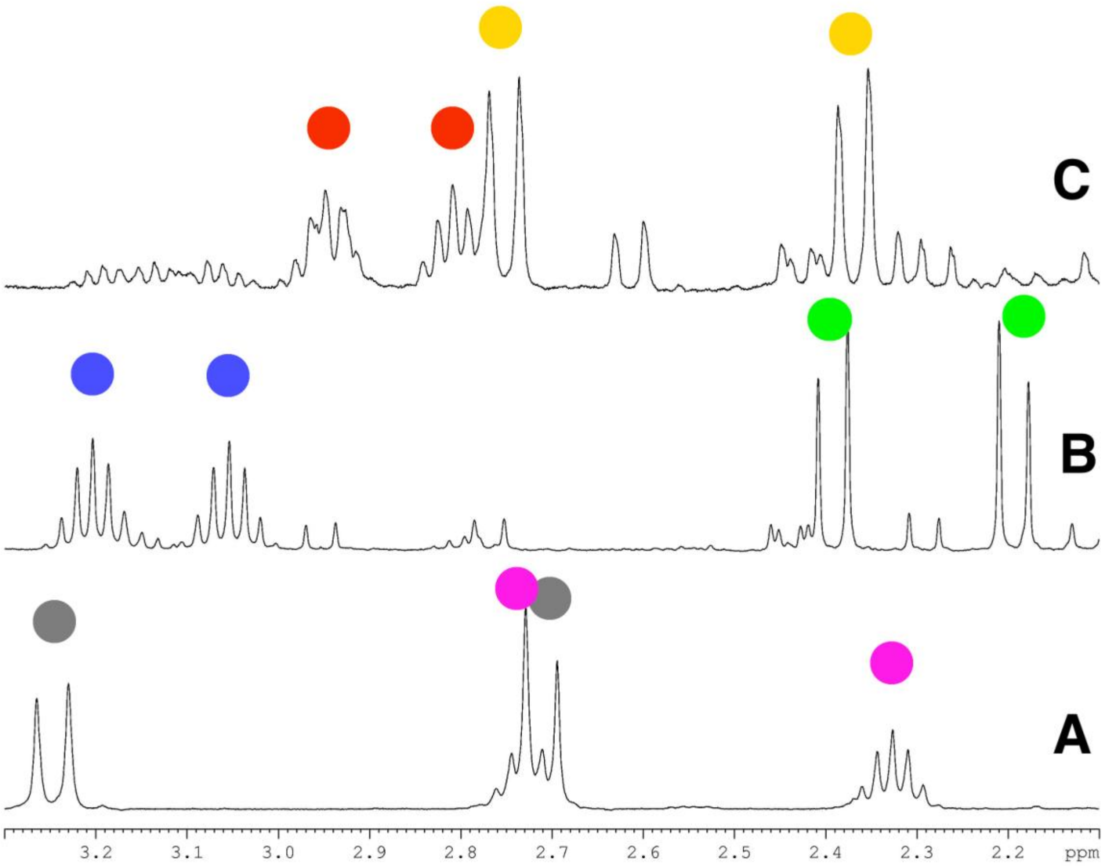

Figure S2: Extracts of the ^{1}H NMR spectra of the starting material 1a (A), the intermediate carbene 2a (B), and the proposed carbene borane 4a (C), all in THF-d_8

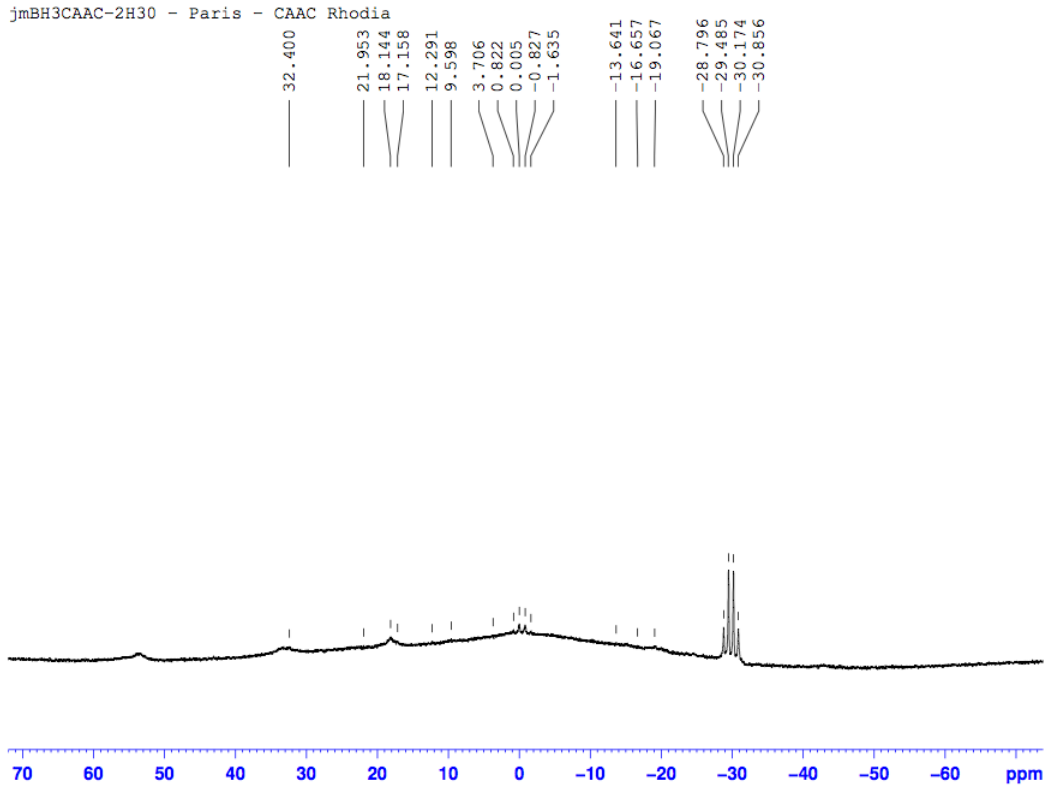

Figure S3: ^{11}B NMR spectrum of CAAC-BH_3 complex 4a after 2.5 h in THF-*d_8*.

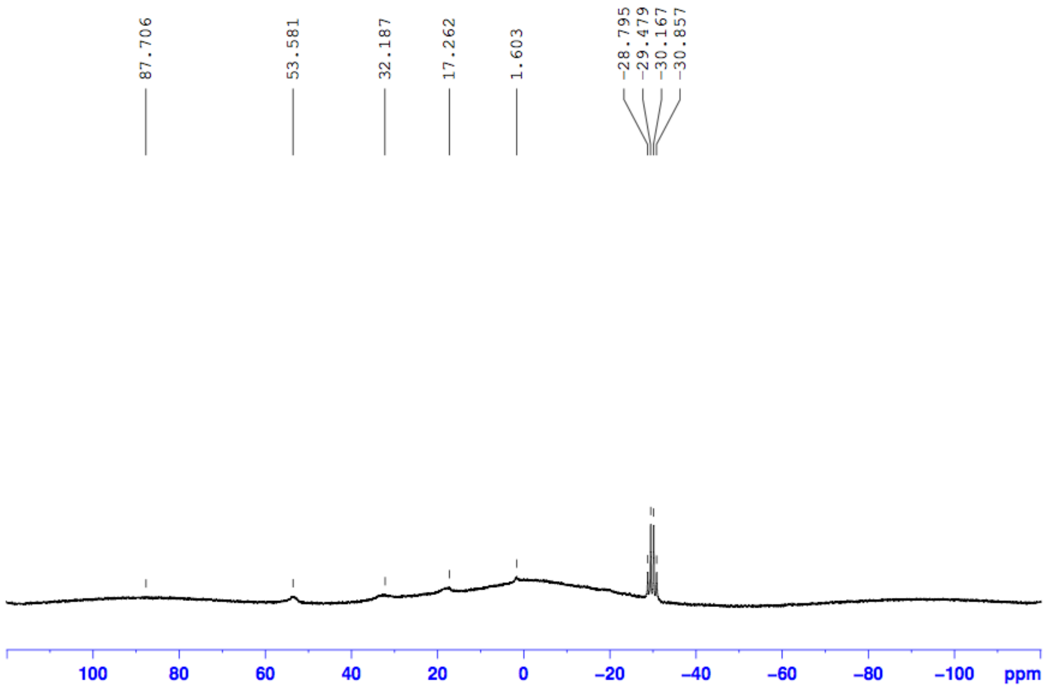

Figure S4: ^{11}B NMR spectrum of CAAC-BH_3 complex 4a after 15 h in THF-*d_8*.
